# Treatment with pCramoll Alone and in Combination with Fluconazole Provides Therapeutic Benefits in *C. gattii* Infected Mice

**DOI:** 10.3389/fcimb.2017.00211

**Published:** 2017-05-24

**Authors:** Jannyson J. Jandú, Marliete C. Costa, Julliana R. A. Santos, Fernanda M. Andrade, Thais F. Magalhães, Márcia V. Silva, Maria C. A. B. Castro, Luanna C. B. B. Coelho, Aline G. Gomes, Tatiane A. Paixão, Daniel A. Santos, Maria T. S. Correia

**Affiliations:** ^1^Departamento de Bioquímica, Universidade Federal de PernambucoRecife, Brazil; ^2^Departamento de Microbiologia, Universidade Federal de Minas GeraisBelo Horizonte, Brazil; ^3^Laboratório de Micologia, Universidade CEUMASão Luís, Brazil; ^4^Núcleo de Enfermagem, Universidade Federal de PernambucoVitória de Santo Antão, Brazil; ^5^Laboratório de Imunogenética, Centro de Pesquisas Aggeu MagalhãesRecife, Brazil; ^6^Departamento de Patologia Geral, Instituto Universidade Federal de Minas GeraisBelo Horizonte, Brazil

**Keywords:** cryptococcosis, immunomodulation, lectins, *Cratylia mollis* lectin, fluconazole, survival

## Abstract

*Cryptococcus gattii* is one of the main causative agents of cryptococcosis in immunocompetent individuals. Treatment of the infection is based on the use of antimycotics, however, the toxicity of these drugs and the increase of drug-resistant strains have driven the search for more effective and less toxic therapies for cryptococcosis. pCramoll are isolectins purified from seeds of *Cratylia mollis*, a native forage plant from Brazil, which has become a versatile tool for biomedical application. We evaluated the effect of pCramoll alone and in combination with fluconazole for the treatment of mice infected with *C. gatti*. pCramoll alone or in combination with fluconazole increased the survival, reduced the morbidity and improved mice behavior i.e., neuropsychiatric state, motor behavior, autonomic function, muscle tone and strength and reflex/sensory function. These results were associated with (i) decreased pulmonary and cerebral fungal burden and (ii) increased inflammatory infiltrate and modulatory of IFNγ, IL-6, IL-10, and IL-17A cytokines in mice treated with pCramoll. Indeed, bone marrow-derived macrophages pulsed with pCramoll had increased ability to engulf *C. gattii*, with an enhanced production of reactive oxygen species and decrease of intracellular fungal proliferation. These findings point toward the use of pCramoll in combination with fluconazole as a viable, alternative therapy for cryptococcosis management.

## Introduction

*Cryptococcus gattii* is a pathogenic fungus that affects mainly immunocompetent individuals. The desiccated yeasts and spores are inhaled and enter the body via the respiratory system, finally infecting the central nervous system (CNS) and causing meningo-encephalitis (Thompson et al., 2012), which frequently has a poor prognosis. Meningitis and meningo-encephalitis are typical in HIV/AIDS and transplant-recipient patients, but also in apparently healthy individuals (Sharon et al., 2012). The main therapies for cryptococcosis treatment caused by *C. gatti* are fluconazole (antifungal azole) and amphotericin B (antifungal polyene; Santos et al., 2012; Reichert-Lima et al., 2016). Fluconazole is used in cases of pulmonary diseases with mild to moderate symptoms. In severe infections, amphotericin B is recommended (associated or not with 5-Flucytosine) followed by a prolonged therapy with fluconazole. Itraconazole, voriconazole, and other azoles are recommended when the use of fluconazole is contraindicated or ineffective (Perfect et al., 2010). Resistance against antifungals (Zhai et al., 2013) enhances clinical failures and increases morbidity and mortality (Ghannoum and Rice, 1999). Furthermore, side effects due to the use of amphotericin B lead to dose-dependent nephrotoxicity frequently associated with increased mortality, requiring monitoring of the renal function of patients. Altogether, these findings increase the demand for new therapies against cryptococcosis.

Previous studies have shown that lectins from plants may have immunomodulatory effects: augmented recruitment and activation of mononuclear and polymorphonuclear leukocytes, development of Th1, Th2, and Th17 response and stimulation of phagocytosis (da Silva and Correia, 2014). *Cratylia mollis* is a native forage plant endemic to the Semiarid Region of Brazil (Caatinga biome), and popularly known as camaratu bean. Four isolectins (Cramoll 1, 2, 3, and 4) can be purified from seeds of the plant. pCramoll (preparation containing isolectins 1 and 4) has binding sites for the specific recognition of glucose/mannose receptors and presents anthelmintic, antiprotozoal, antitumoral, healing, and immunomodulatory effects (Maciel et al., 2004; Melo et al., 2011; da Silva et al., 2014). Focusing on immunoregulatory properties, the aim of this study was to evaluate the immunomodulatory effect of pCramoll in a murine model of infection by *C. gattii*. Our results revealed that this lectin is able to increase survival, decrease fungal burden in organs and reduce the morbidity of cryptococcosis.

## Materials and methods

### pCramoll

*C. mollis* seed extract (10% w/v prepared in 0.15 M NaCl) was fractionated using ammonium sulfate (40–60% w/v) and the fraction obtained was submitted to affinity chromatography in a Sephadex G-75. The pCramoll preparation was bioselectively eluted with 0.3 M of D-glucose in 0.15 M NaCl, dialyzed against 0.15 M NaCl for 24 h and subsequently lyophilized (Correia and Coelho, 1995) and diluted in PBS to analyze protein concentration, as determined by the BCA kit (Thermo Fisher Scientific Inc., Waltham, MA, USA).

### Phagocytosis assay, intracellular proliferation rate (IPR), measurement of ROS, and NO production by macrophages

Bone marrow cells were isolated as described previously (Weischenfeldt and Porse, 2008; de Souza et al., 2016). Femurs and tibias from mice were removed, disinfected by immersion in 70% ethanol and the ends of each bone were cut. Both bones were flushed with 5 mL of cold RPMI 1640 (HyClone, LGC Biotecnologia) using a 5-mL syringe and a 25-gauge needle. The cell suspension was centrifuged for 5 min at 1,200 rpm at 4°C and washed once with cold RPMI. Then, bone marrow cells were counted using a hemocytometer and the concentration was adjusted to 2 × 10^6^ cells/mL for plating on tissue culture–treated petri dishes in BMM medium (RPMI supplemented with 30% L929 growth conditioning media, 20% bovine fetal serum [Gibco], 2 mM glutamine [Sigma], 25 mM HEPES pH 72, 100 units/mL of penicillin-streptomycin [Life Technologies]). Fresh media were added every 48 h. Bone marrow–derived macrophages (BMDMs) were collected on day 7 and used for subsequent experiments. Under these conditions, macrophages/monocytes progenitors will proliferate and differentiate into a homogenous population of mature BMDMs, obtained >90% pure live macrophages (Zhang et al., 2008; Bouwman et al., 2014; Bhattacharya et al., 2015; Heung and Hohl, 2016). For all analyses, the cells were infected with L27/01 yeasts cells (growth on Sabouraud Dextrose Agar (SDA) medium, for 48 h at 35°C) opsonized with 10% murine serum in RMPI suspension (0.4 × 10^6^ cells/mL), at the proportion of 5 macrophages: 1 *C. gattii*, under cell treatment with 1 or 5 μM pCramoll and incubated for 3 or 24 h at 37°C under 5% CO_2_.

The phagocytic index was performed using 24-well plates and a single coverslip 13 mm in diameter. After 3 h, these coverslips containing adherent cells were removed, stained with Giemsa and the index was calculated as the percentage of cells with internalized *C. gattii* 24 h post-infection (Santos et al., 2014). Images from the phagocytosis assay were obtain using a Nikon COOLPIX 4500 camera coupled to a Nikon ECLIPSE E200 microscope with 100 x of magnification and analyzed using the ImageJ Protocol (http://rsbweb.nih.gov/ij/); the cell area (average of 100 cells per coverslip) was measuring as described by Baviskar (2011).

The intracellular proliferation rate (IPR) assay was performed as previously described (Ma et al., 2009) with modifications. Non-internalized yeast cells in the supernatant were taken from the wells and the adherent phagocytes were washed with 200 μL PBS. These macrophages were lysed at 3 and 24 h with 200 μL of cold, sterile, distilled water and incubated for 30 min, then 100 μL was collected and plated on SDA medium, and the viable yeast cells were counted. The IPR was calculated as the quotient of the intracellular yeast cell numbers at 24 h (the point in time which featured the maximum intracellular yeast number) and 3 h.

The supernatant of phagocytosis assay was used for measurement of NO production using the Griess assay. The nitrite concentration was quantified by extrapolation from a sodium nitrite standard curve, determined at 540 nm with a microplate reader (Benchmark Plus, Bio-Rad, CA, US).

Macrophages/well in RPMI-1640 without phenol red (Sigma-Aldrich) were also infected with *C. gatti* (same conditions that phagocytosis assay), and the supernatant was used for ROS quantification using 2-7-dichlorofluorescein diacetate (DCFH-DA; Invitrogen, Life Technologies, Carlsbad, CA, USA) was used and the fluorescence was measured with a fluorometer (Synergy 2 SL Luminescence Microplate Reader; Biotek) the positive control was hydrogen peroxide at 10 μM (Ferreira et al., 2013).

### Effect of pCramoll and fluconazole in the *Cryptococcus gattii* infection model

The protocol of the animal studies was approved by the Comissão de Ética no Uso de Animais (CEUA) at the Universidade Federal de Minas Gerais (Protocol 310/2014). All mice were housed in clean cages, with food and water *ad libitum*. The controlled environment was set to a 12 h light/dark cycle at 23°C. Mice C57BL/6, 5–6 weeks old (*n* = 6/group) were anesthetized by intraperitoneal (i.p.) injection of ketamine hydrochloride (60 mg.kg^−1^) and xylazine (10 mg.kg^−1^) in PBS, and then inoculated by an intratracheal infection with 30 μL of 10^4^ CFU/animal of *C. gattii*, L27/01 strain. Intratracheal infection with the *C. gattii* L27/01 strain (GVII molecular type) has been previously shown in other works (Santos et al., 2014; Ferreira et al., 2015).

Initially, infected mice were intraperitoneally (i.p.) treated every 10 days with 1, 250, or 500 μg of lectin alone (the first dose was administered 1 day before intratracheal infection). For the group treated with the combination fluconazole (20 mg.kg^−1^) and pCramoll at 1, 250, or 500 μg (the fluconazole was daily administered and every 10 days the association was used), or the mice were treated daily with fluconazole alone (20 mg.kg^−1^) for survival monitoring. The untreated group (NT), and not infected group (NI) were inoculated with PBS. All animals were monitored twice daily for survival and behavior parameter analysis (SHIRPA protocol). The SHIRPA protocol analyzes the behavioral and functional assessment of neurological diseases (Rogers et al., 1997). The tasks are grouped into five functional categories: neuropsychiatric state, motor behavior, autonomic function, muscle tone and strength, and reflex and sensory function (Santos et al., 2014; Costa et al., 2016). All mice were examined daily and the score for each functional category was calculated as the total of the evaluated parameters according to Lackner et al. (2006) and Pedroso et al. (2010) using the EpiData 3.1 software. The Table S1 describes all the parameters analyzed in the SHIRPA protocol.

Furthermore, other groups of mice were i.t. infected and treated with pCramoll at 1 μg per mouse (the concentration that provided the best results in the survival curve), combined or not with fluconazole to obtain lungs and brain at 15 or 35 days post-infection (d.p.i). The animals were euthanized by cervical dislocation under anesthesia. The lungs and brain were removed for determining the colony-forming units (CFU) as described previously (Santos et al., 2014). For this, the organs were homogenized with sterile PBS and plated in SDA medium. Specifically, 100 or 50 mg of lung tissue was homogenized with 1 or 0.5 mL of extraction solution of protein, containing PBS (pH 7.0) and anti-proteases (0.1 mM PMSF, 0.1 mM benzethonium chloride, 10 mM EDTA, 20 Kallikrein inhibitor units of aprotinin A, all purchased from Sigma-Aldrich and 0.05% tween 20). The samples were centrifuged for 10 min at 3,000 × g, at 4°C and the supernatant was frozen at −20°C and utilized for cytokine analysis. The levels of IL-10 and IL-6 were determined using commercially available antibodies according to the manufacturer's instructions (R&B Systems, Minneapolis, MN, USA) and the levels of IFN-γ and IL-17A were measured by cytometric bead array (BD Biosciences, San Jose, CA, USA). Also, the bronchoalveolar lavage fluid (BAL) was obtained and centrifuged at 1,200 rpm for 5 min at 4°C. The cell pellet was suspended in 3% albumin solution (100 μl). A 10 ul aliquot of albumin solution containing the cells washed was diluted in 10 uL of Türck for cell count totals in a Neubauer chamber with an optical microscope at 40x magnification. For differential counts, smears were made in cellspin (CT-2000, CIENTEC), stained using the Giemsa method and cells were quantified by morphological criteria for the distinction of cellular types (mononuclear and polymorphonuclear) and the results were grouped according to the percent content of these two cells type (Maxeiner et al., 2007).

### Myeloperoxidase (MPO) and N-acetylglucosaminidase (NAG) activities

The infiltration of neutrophil in the lungs was indirectly measured by the assay of myeloperoxidase activity (MPO) according to Costa et al. (2016), by measuring the change in optical density (OD) using 3,3′,5,5′-Tetramethylbenzidine (TMB) (Sigma-Aldrich). The absorbance reading was taken at 450 nm in a spectrophotometer. For N-acetylglucosaminidase activity (NAG), 2,24mMp-nitrophenyl-N-acetyl-β-D-glicosaminide (Sigma-Aldrich) was used and absorbance was determined at 400 nm (Baltazar et al., 2014).

### Histopathology

The lungs were removed during necropsy and immediately fixed in buffered 10% formalin (v/v). The tissue was embedded in paraffin, and the sections were stained with hematoxylin and eosin (Sigma) and examined under light microscopy.

### Statistical analysis

Statistical analysis of all data were performed using GraphPad Prism version 5.0 with *p* < 0.05 considered significant. The survival curve was plotted by Kaplan-Meier analysis and the results were analyzed using the log rank test, for behavior parameters the area under the curve was analyzed. Also, the results of the phagocytosis assay, IPR, measurement of ROS and NO production by macrophages, MPO and NAG activity, and quantification of cytokines were analyzed by analysis of variance (ANOVA) followed by Dunn's Multiple Comparison Test and the Student's *t*-test.

## Results

### Preliminary tests of toxicity and antifungal activity of pCramoll

Previously, the pCramoll toxicity analysis in *Caenorhabditis elegans* model was performed complementarily. Non-toxicity was verified in these worms (data not shown) and confirms the non-cytotoxicity of the pCramoll concentration used in this study and published previously by da Silva et al. (2014). Subsequently, we investigated the antifungal activity of pCramoll against *C. gattii* by screening and determining the minimum inhibitory concentration (MIC). Neither of the concentrations tested (0.93–120 μg/mL) were able to inhibit fungal growth (data not shown). Indeed, no interaction between pCramoll and fluconazole was obtained by the checkerboard test (data not shown).

### pCramoll increases phagocytosis and fungicidal activity of bone marrow-derived macrophages (BMDM)

**Figure 1:**
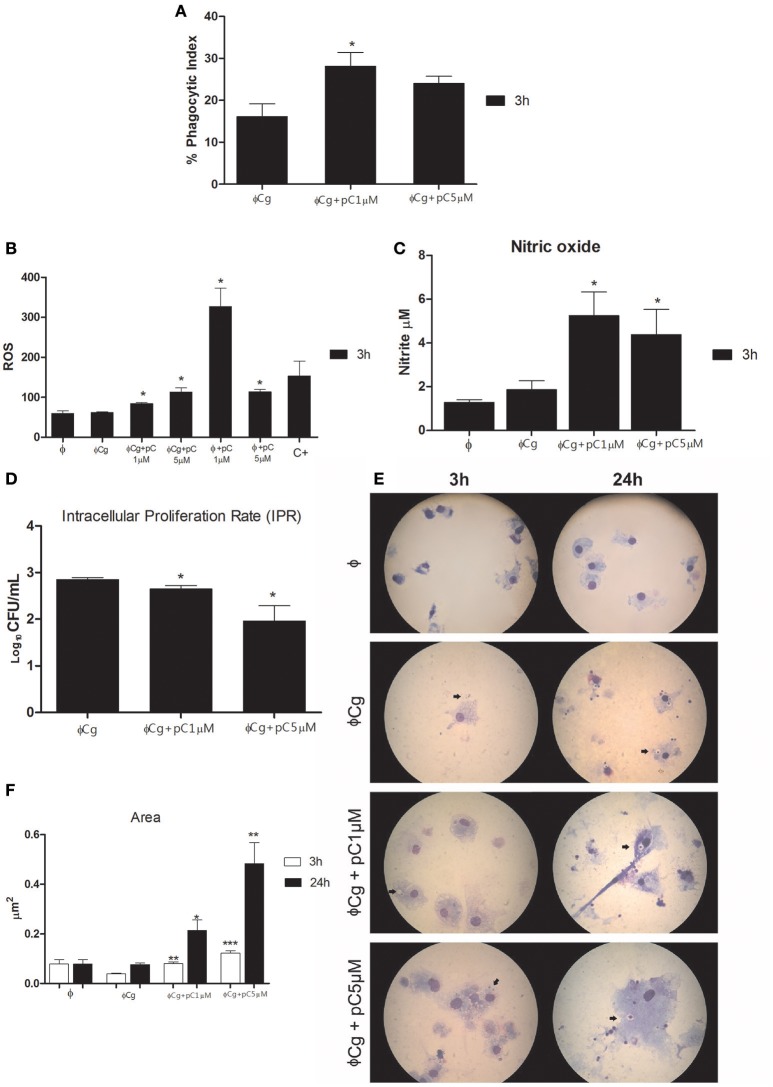
**Immunomodulatory and fungicidal effect of pCramoll in murine bone marrow macrophages infected with ***Cryptococcus gattii***. (A)** After 3 h of incubation, the phagocytic index increased under pCramoll treatment to 1 μM; **(B)** After 3 h of incubation, treatment with pCramoll enhances ROS production in macrophages infected with *C. gattii*, in a concentration-dependent manner. **(C)** Oxide Nitric production after 3 h after incubation; **(D)** Intracellular Proliferation Rate after 24 h of incubation; **(E)** Macrophage morphology after 24 h of incubation with *C. gattii*, showing *C. gattii* phagocytosis (arrows). Cell cultures were observed under 100 × optical zoom and 10 fields per coverslip. **(F)** Area of macrophages stimulated with lectins and subsequently infected with *C. gatti*. *p < 0.05; ^**^p < 0.01; ^***^p < 0.005 (Difference between treatment groups and infected control); Φ, Uninfected macrophages, ΦCg, *C. gattii-*infected macrophages; ΦpC, Uninfected macrophages treated with pCramoll; C+ 10 μM Hydrogen peroxide. Data represent the means ± SE from two independent experiments consisting of ten replicate assays.

pCramoll at 1 μM increased the phagocytic index after 3 h of incubation (Figure 1A), compared with the untreated group. At the same time, the levels of ROS were increased in the presence of pCramoll at 1 and 5 μM, both for infected and uninfected macrophages (Figure 1B), especially with pCramoll at 1 μM with significant increase in the production of nitrosative species (Figure 1C). This increase in ROS levels was associated with a reduced IPR (Figure 1D). Another interesting observation was the presence of macrophage aggregates, vacuoles and increased expansion of the cells (area) in the presence of increasing concentration of pCramoll (Figure 1E). Bone marrow-derived macrophages stimulated with pCramoll at 1 and 5 μM showed, 3 h post-incubation, 0.080 and 0.197 μm^2^ of area, respectively, while at 24 h: 0.214 and 0.483 μm^2^, respectively. There was an increase of up to 5 × in cell area compared with untreated macrophages infected with *C. gattii* after 3 h and to 7 × after 24 h of incubation (Figure 1F).

### pCramoll and fluconazole increase the survival and improve the behavior of animals infected with *C. gattii*

**Figure 2:**
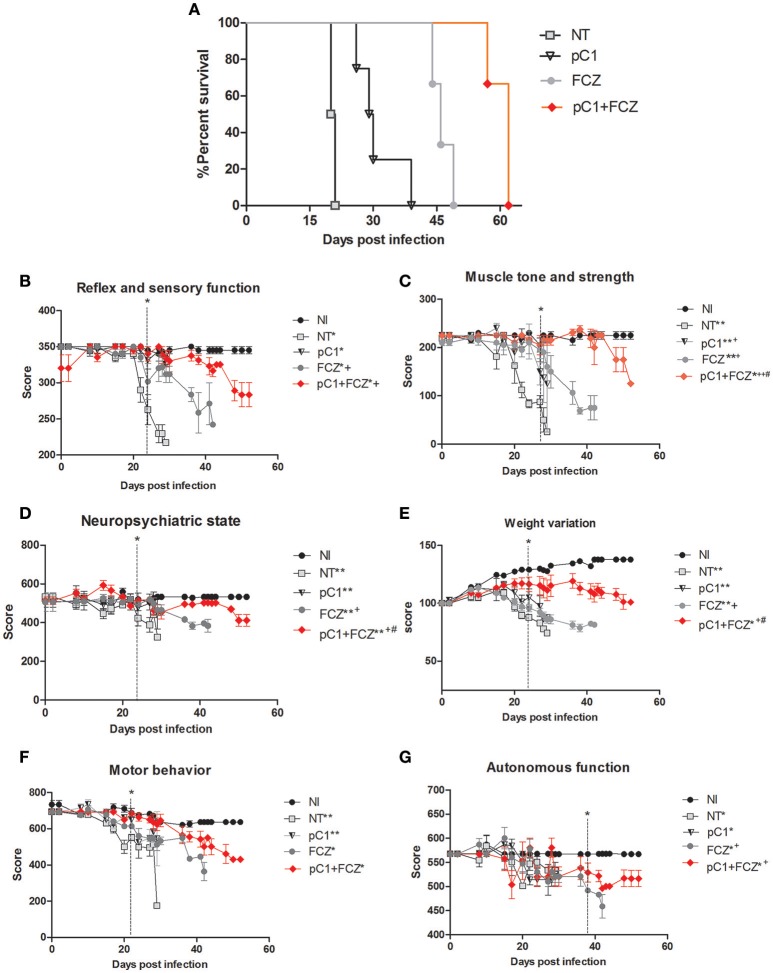
**Survival curve and behavioral profile evaluation of mice infected with ***Crytococcus gattii*** and subjected to different treatments. (A)** Six mice per group were inoculated with 10^4^ cells of L27/01 strain by intratracheal line inoculation and treated with fluconazole at 20 mg.kg^−1^ (FCZ), pCramoll at 1 ug (pC1), or the combination (pC1 + FCZ). Animals treated by pC1 + FCZ had an average increase of over 34.8% in survival compared to those treated with FCZ alone ^*^p < 0.05. **(B–G)** Five animals per group were submitted to the SHIRPA Protocol. There was no behavioral difference between uninfected mice (NI) and those treated with pC1 alone, which did also not influence the survival of mice. Untreated mice (NT); ^*^p < 0.05; ^**^p < 0.001 (difference when compared to NI); ^+^*p* < 0.5; ^++^*p* < 0.001 (difference when compared to NT); ^#^*p* < 0.05 (difference between FCZ and combination). Data represent the means ± SE from three independent experiments.

An increase in survival of animals treated with pCramoll was observed. The untreated group (NT) showed a median survival (MS) of 20 days (Figure 2A), while the MS for groups treated with the lectin at 500, 250, and 1 μg, were 30.5 (*p* < 0.05; data not shown), 30 (*p* < 0.01; data not shown) and 29.5 (*p* < 0.01) days (Figure 2A), respectively, an increase of 50% (approximately) for all doses tested, and an independent dose response. pCramoll at 500, 250, and 1 μg was also tested in uninfected animals and no change in the survival/behavior of animals compared to NI mice was found (data not shown). When combined with fluconazole, the influence of lectins on survival was dose dependent. The group treated with fluconazole alone showed an MS of 46 days, while the groups where pCramoll was combined with the antifungal demonstrated a MS of 51 days for 500 μg of pCramoll (data not shown), 58 days for 250 μg of pCramoll (data not shown) and 62 days for 1 μg of pCramoll (*p* < 0.05; Figure 2A). Based on these results, the dose of 1 μg of pCramoll combined or not with fluconazole was used in the further experiments. Indeed, the cellular polysaccharide extracted from strain L27/01 (PSC) of *C. gattii* was associated with pCramoll and fluconazole, and were tested in our model. The administration of PSC only hurried the death of infected animals (MS of 17 days), compared to the NT group (20 days). The same was observed where PSC was associated with pCramoll and fluconazole (MS of 43 days), compared to the FCZ-treated group (MS of 46 days), where the animals had previously succumbed (data not shown).

The behavior analysis of basic functions such as sensory-reflex (Figure 2B), muscle tone and strength (Figure 2C) demonstrated improved behavior by day 20 post-infection when animals were treated with pCramoll, fluconazole and the combination compared to the untreated group (NT). Moreover, the combination is significantly more efficient than fluconazole alone (*p* < 0.05). A similar behavioral profile was observed for parameters involved in the neuropsychiatric state (Figure 2D) and body weight (Figure 2E), which improved by day 22 day post-infection (*p* < 0.05) for all the treated groups, but with better performance for groups treated with the combination (*p* < 0.05). There was no difference between the treated and untreated groups for motor behavior (Figure 2F). Finally, as shown in Figure 2G, autonomic function was only improved when the combination was used (*p* < 0.05).

### The combination pCramoll + fluconazole reduces fungal burden and increases inflammatory response

**Figure 3:**
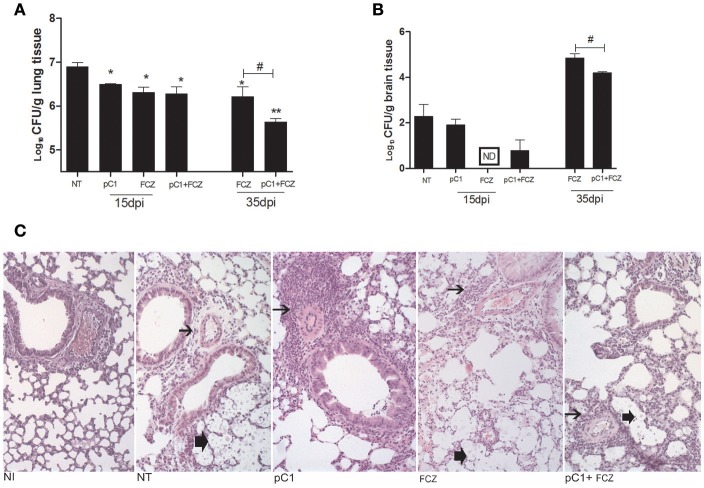
**pCramoll in combination with fluconazole shows increased survival and reduced fungal burden in the lungs and brain of ***Cryptococcus gattii***-infected mice**. Six mice per group were inoculated with 10^4^ cells of the L27/01 strain by intratracheal line inoculation and given the combination treatment, analyzed at 15 and 35 dpi: **(A,B)** Colony-forming Units (CFU) recovered from brain and lungs, respectively, at 15 and 35 days post infection; **(C)** Histological sections of lung tissue stained with H&E at 200 ×, 15 days post inoculation. Non-infected mice showed normal histology. Mice treated with pC1 showed more prominent perivascular inflammatory infiltrate (thin arrow) and less yeasts (thick arrow) in alveolar space than untreated, infected mice. Mice from FLZ or pC1+ FLZ showed moderate inflammation. Uninfected animals (NI), infected untreated control (NT), groups treated with pCramoll at 1 μg (pC1), Fluconazole (FCZ), ND (not recovered CFU). ^*^p < 0.05; ^**^p < 0.01 (difference between treated groups and NT); ^#^*p* < 0.05 (difference between FCZ alone and combination, at 35 dpi). Data represent the means ± SE from three independent experiments consisting of triplicate assays.

All the treatments reduced the fungal burden in the lungs after 15 dpi. At 35 days post infection, fluconazole combined with pCramoll was better than fluconazole alone in reducing pulmonary fungal burden (*p* < 0.05; Figure 3A). In brain tissue, only the use of fluconazole reduced the fungal burden after 15 dpi (Figure 3B), however, the combination (fluconazole + pCramoll) was able to reduce cerebral fungal burden significantly when compared with fluconazole alone after 35 dpi (*p* < 0.05). Histological analysis of lung tissue from untreated infected mice at 15 dpi showed numerous yeasts in the pulmonary parenchyma, mild perivascular infiltrate and alveolar thickening. Moderate to intense perivascular inflammation was observed in lung tissue from pCramoll, and pCramoll + fluconazole groups. Lungs from treated groups showed less yeast in alveolar space than the untreated group (Figure 3C).

**Figure 4:**
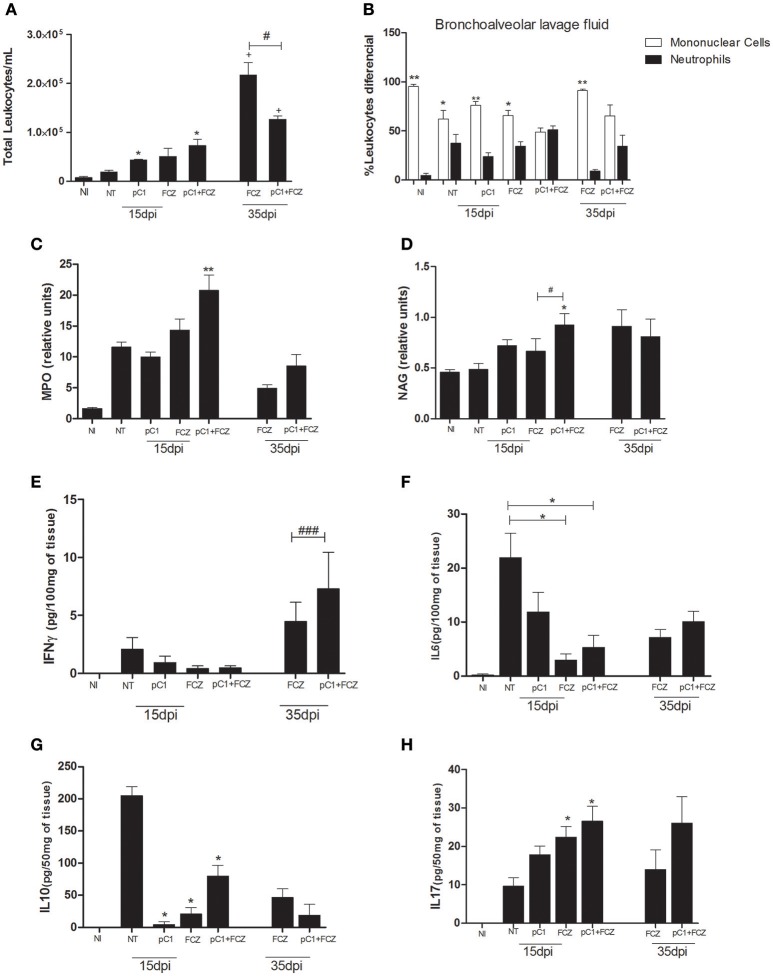
**Inflammatory response of pCramoll in combination with fluconazole in ***Cryptococcus gattii***-infected mice, analyzed at 15 and 35 dpi: (A,B)** Total and differential count of leukocytes (neutrophils and mononuclear cells) in the bronchoalveolar lavage, respectively; at 15 and 35 days after infection; **(C,D)** Myeloperoxidase and N-acetylglucosaminidase activities in lungs of infected animals; **(E–H)** Levels of cytokines IFN-γ, IL-6, IL-10, and IL-17A in lungs. Uninfected animals (NI), infected untreated control (NT), groups treated with pCramoll at 1 μg (pC1), Fluconazole (FCZ), ^*^p < 0.05; ^**^p < 0.01 (difference between treated groups and NT); ^#^*p* < 0.05 (difference when comparing treated groups); ^*###*^*p* < 0.005 (difference between FCZ alone and combination, at 35 dpi); ^+^*p* < 0.5 (difference between 15 dpi and 35 dpi). Data represent the means ± SE from three independent experiments consisting of triplicate assays.

Regarding the inflammatory infiltrate in the bronchoalveolar lavage fluid (Figure 4A), we verified a predominance of mononuclear cells for all groups except the one treated with the combination, for which there was a balance between mononuclear and polymorphonuclear cells, at both 15 and 35 dpi (Figure 4B). Additionally, MPO (Figure 4C) and NAG (Figure 4D) activities were performed in order to confirm the presence of neutrophils and macrophages, respectively, where the combination demonstrated higher activities of both enzymes at 15 dpi.

The levels of IFN-γ (Figure 4E) were higher for the group treated with the combination at 35 dpi. The levels of IL-6 (Figure 4F) and IL-10 (Figure 4G) were augmented in the NT group. An increase in IL-17A (Figure 4H) was verified for the combination and for fluconazole alone at 15 dpi.

## Discussion

We observed complementarily that pCramoll did not show toxicity in the *C. elegans* model, corroborating previously published *in vitro* cytotoxicity data in mammalian cells (Melo et al., 2010, 2011; da Silva et al., 2015). In addition, pCramoll did not present antimicrobial action against *C. gatti*, an activity rarely found in the legume lectin domain (Dias et al., 2015). However, a few studies have shown the immunomodulatory potential of plant lectins to combat bacterial and fungal infections (Alegre-Maller et al., 2014), in this context pCramoll influences the inflammatory responses against *C. gatti*.

pCramoll increased the phagocytosis of *C. gattii* and the production of ROS and NO, augmenting the fungicidal activity of macrophages. Furthermore, this effect was observed in the cellular morphology of infected macrophages. Previously, the immunoregulatory influence of pCramoll has been portrayed, showing increased phagocytosis of *S. aureus*, as well as enhancement of NO, ROS, and pro-inflammatory cytokines (da Silva et al., 2014). Phagocytosis is the main mechanism for fungal elimination (Leopold et al., 2016), and is associated with increased survival of infected animals. Artin M (another mannose-specific lectin, like pCramoll) demonstred that phagocytosis was also the mechanism responsible for *Candida albicans* elimination, reducing the fungal burden in target organs and increasing animal survival (Loyola et al., 2012).

The murine model used in this study mimics the natural route of infection by *C. gattii*, as confirmed by the results of fungal burden and alterations in histopathology and behavior. pCramoll alone, as well as when combined with fluconazole, enhanced the survival of animals and improved the behavioral alterations caused by neuro-cryptococcosis. pCramoll in association with fluconazole decreased distinct alterations in related parameters, such as sensory-reflex function, the neuropsychiatric state, muscle tone, and strength, as well as body weight. In the course of the disease, these clinical manifestations are determinants for neurological sequelae and death (Chen et al., 2012).

The pCramoll and fluconazole treatments decreased CFU in the lungs, at 15 dpi. Not only does this association enable a significant reduction in CFU, it is also more effective than the use of fluconazole alone in treating pulmonary cryptococcosis. Although yeasts were recovered from the brain of animals treated with the combination, they were not found in animals treated only with fluconazole. This may be due to pCramoll not crossing the blood-brain barrier (Patricio et al., 2011) and not directly acting on the CNS. Meanwhile, fluconazole was present at higher concentration in the CNS after 14 days of use, reducing CFU in the brain (Mendes et al., 2010). Furthermore, pCramoll also stimulates *C. gattii* phagocytosis, increasing the early combat of the pathogen in the lungs. However, some yeasts have the capacity to survive in phagolysosomes, enabling transmigration of the fungus (Charlier et al., 2009). This phenomenon reduces fungal permanence in lung tissue, inhibiting strategies of fungal adaptation, avoiding cellular modification which inhibit the phagocytosis, and more virulent growth, like giant, fluconazole-resistant cells (Kronstad et al., 2011).

Interestingly, 35 days post-infection, there was a significant decrease in CFU in the lungs and brain in animals treated with the combination compared to the use of fluconazole alone. Ongoing use of the combination resulted in better neurological and behavioral conditions. This therapeutic characteristic is very important because previous studies have demonstrated that most patients infected with *C. gattii* presented CNS cryptococcosis, especially with meningitis 64–76% (Chen et al., 2000, 2012). The reduced number of yeasts in the brain improves the general conditions of mice, as confirmed by the results shown in the survival curve.

Infections with *C. gattii* leads to suppression of the host's immune responses, including decreased leukocyte recruitment and pro-inflammatory cytokine production (Wright et al., 2002; Leopold et al., 2016). On the other hand, the therapy tested in this work has shown that, at 15 dpi, the animals treated with pCramoll alone increase leukocyte migration with a predominance of monocytes to macrophages contained in the BAL, although with less expression of inflammatory cytokines IFN-γ, IL-4, and TNF-α in the lungs (data not shown). The recruitment of these cells and their presence in the inflammatory response are crucial for immunological responses and pathogen depletion, mainly by phagocytosis (Melo et al., 2010).

At 15 dpi, when pCramoll was combined with fluconazole, despite the large inflammatory infiltrate, the leukocyte content was balanced (neutrophils and macrophages), with lower Th1 cytokine expression (IFN-γ) as well as IL-10 auto-regulator. This is very important because the highly detrimental effect of IL-10 in cryptococcal infection models, as shown in NT mice, plays a major role in downregulating cryptococcal clearance (Olszewski et al., 2014). The same way as at 15 dpi, the high IL-17A production was observed for the combination. This inflammatory profile is not limited to the Th1/Th2 balance polarization against the infection, this response has been credited to IL-17A cytokine, which is known to have a role in cellular recruitment (Kolls and Lindén, 2004; Steinman, 2007) and contribute to anti-cryptococcal protection (Voelz et al., 2009).

Up to 35 dpi, an increase of IFN-γ was observed in the lungs of mice treated with the combination, followed by IL-17A production, however with lower inflammatory infiltrate compared to the fluconazole group. The early decrease of IL-10 as seen in the groups treated with pC and their combination enhances IFN-γ and IL-17A effector responses and promotes fungal clearance in mice with cryptococcal lung infection (Murdock et al., 2014a). Previous studies have shown that pCramoll lectin stimulates Th17- and modulate the Th1-type cytokines production (Oliveira et al., 2013). The influence of the Th17 pathway in resistance to fungal pathology is a prospective finding in experimental Arpergilosis and Candidiasis studies (Romani and Puccett, 2006; Zelante et al., 2009), while the role of Th1-type cytokines has also been demonstrated (Voelz et al., 2009; Gibson and Johnston, 2015). Moreover, the moderate inflammation is fundamental to the mechanism of tissue repair, associated with antifungal pathways, as seen in associated response mechanisms of IL17/IL10 cytokines (Zizzo and Cohen, 2013; Murdock et al., 2014b). Lower/moderate inflammation contributes to better neurological conditions.

Therefore, pCramoll is an immunomodulatory lectin without antifungal activity but its combination with fluconazole increases the survival of animals with cryptococosis, improving aspects of morbidity present in disease progression.

## Conclusion

Immunotherapy with lectins in the treatment of cryptococcosis has not been reported, making this work pioneering. The immunoregulatory effect of pCramoll increases the survival of animals and improves clinical signs. Combination with fluconazole was able to reduce fungal burden in the lungs and brain of infected animals, increase *C. gattii* phagocytosis and produce higher levels of ROS. As such, the combination thereby represents a promising alternative in the treatment of cryptococcosis.

## Author contributions

JJ: performed plant collection, biochemical, antimicrobial, and immunologycal assays, analysis and wrote the paper; JS: contributed in the experimental developing with animals; MC: permed experimental procedures *in vivo, in vitro*, as well as theoretical aspects of the paper. FA: participated plant collection, and purification process of the lectin. TF: contributed in experimental procedures with animals. AG: Contributed in the histological analysis. MS, LC: Scientific and structural support for research development. MB: Participated in the analysis of inflammatory factors by flow cytometry. TP: collection, and purification process of the lectin.Contributed in the histological analysis and theoretcal aspects. DS: Coordinated the project, supervised all development of this work, such theoretical and experimental aspects this research. MC: Coordinated the project. Scientific and structural support for research development.

### Conflict of interest statement

The authors declare that the research was conducted in the absence of any commercial or financial relationships that could be construed as a potential conflict of interest. The reviewer CPT and handling Editor declared their shared affiliation, and the handling Editor states that the process nevertheless met the standards of a fair and objective review.
