# Supplementary material for: Treatment with pCramoll Alone and in Combination with Fluconazole Provides Therapeutic Benefits in C. gattii Infected Mice
Source: Front Cell Infect Microbiol. 2017 May 24;7:211. doi: 10.3389/fcimb.2017.00211 (PMC5442327; doi:10.3389/fcimb.2017.00211)
Supplement: Supplementary file 1 [file Table1.DOC]

**The lectin pCramoll acts as a therapeutic adjuvant for the treatment of murine cryptococcosis caused by *Cryptococcus gattii***

Jannyson José Braz Jandú^a^, Julliana Ribeiro Alves dos Santos^e^, Fernanda Andrade^a^, Marliete Carvalho da Costa^b^, Thais Furtado Ferreira Magalhães^b^, Márcia Vanusa da Silva^a^, Maria Carolina Accioly Brelaz de Castro^c,d^, Luanna Cassandra Breitenbach Barroso Coelho^a^, Aline Gonçalves Gomes^f^, Tatiane Alves Paixão^f^, Daniel AssisSantos^b^, Maria Tereza Santos Correia^a*^

^a^Departamento de Bioquímica, Universidade Federal de Pernambuco, Pernambuco, Brasil

^b^Departamento de Microbiologia, Universidade Federal de Minas Gerais, Belo Horizonte, Minas Gerais, Brasil

^c^Núcleo de Enfermagem, Universidade Federal de Pernambuco, Pernambuco, Brasil

^d^Laboratório de Imunogenética, Centro de Pesquisas Aggeu Magalhães, Pernambuco, Brasil

^e^Laboratório de Micologia, Universidade CEUMA (UNICEUMA), São Luís, Maranhão, Brasil

^f^ Departamento de Patologia Geral, Instituto Universidade Federal de Minas Gerais, Belo Horizonte, Minas Gerais, Brazil

*Corresponding author.

Tel.: +55 8121268540;

fax: +55 8121268576.

email address: mtscorreia@gmail.com

**Table S1. SHIRPA (evaluated parameters**)

| **Functional categories** | **Parameters**  (Lackner et al. 2006; Pedroso et al. 2010; Santos et al., 2014; Costa et al., 2016). |
| --- | --- |
| Muscle tone and strength | Grip strength, body tone, limb tone, abdominal tone |
| Motor behavior | Body position, tremor, locomotor activity, pelvic elevation, gait, tail elevation, trunk curl, limb grasping, wire maneuver, negative geotaxis |
| Neuropsychiatric state | Spontaneous activity, transfer arousal, touch escape, positional passivity, biting, fear, irritability, aggression, vocals |
| Autonomous function | Respiration rate, defecation, urination, palpebral closure, piloerection, skin color, heart rate, lacrimation, salivation |
| Reflex and sensory function | Startle response, visual placing, pinna reflex, corneal reflex, toe pinch, righting reflex |
